# How to move towards One Health surveillance? A qualitative study exploring the factors influencing collaborations between antimicrobial resistance surveillance programmes in France

**DOI:** 10.3389/fpubh.2023.1123189

**Published:** 2023-07-11

**Authors:** Clémence Bourély, Léo Rousset, Mélanie Colomb-Cotinat, Lucie Collineau

**Affiliations:** ^1^French Ministry of Agriculture and Food, General Directorate for Food, Animal Health Unit, Paris, France; ^2^Epidemiology and Support to Surveillance Unit, French Agency for Food, Environmental and Occupational Health and Safety (ANSES), University of Lyon, Lyon, France; ^3^Claude Bernard University Lyon 1, Lyon, France; ^4^VetAgro Sup, Marcy-L'Étoile, France; ^5^Direction des Maladies Infectieuses, Santé Publique France, Saint-Maurice, France

**Keywords:** One Health surveillance, collaboration, integration, antimicrobial resistance, antibiotics, qualitative research

## Abstract

**Introduction:**

Antimicrobial resistance (AMR) is a major public health issue, against which international organisations and governmental bodies call for integration between surveillance programmes involved in human, animal, and environmental sectors. Collaborations are the primary feature of integration and deserve to be supported. However, little is known about the factors that can foster collaborations between surveillance programmes. This study aimed to provide a better understanding of the factors for setting-up collaborations between AMR surveillance programmes in France.

**Methods:**

We performed a qualitative study based on 36 semi-structured interviews with programmes’ coordinators and 15 with key-informant experts involved in AMR surveillance.

**Results:**

The implementation of collaboration between sectors was multifactorial: we identified 42 factors grouped into six categories (i.e., characteristics of the overall AMR surveillance system, features of the collaborating programme, profile of the actors involved, characteristics of the collaboration itself, broader context, and AMR research activities). Collaborations were mainly fostered by good interpersonal relationship between actors, their interest in transdisciplinary approaches and the benefits of collaboration on the programmes involved. Limited resources and the complexity of the AMR surveillance system hindered collaboration. Paradoxically, coordinators generally did not perceive collaborations as a resource-pooling tool since they generally set them up only after consolidating their own programme.

**Discussion:**

Since most factors identified were not specific to AMR, these results can be useful for other collaborative surveillance system. Ultimately, they provide a better understanding of stakeholders’ motivations and influences driving collaboration, and can help researchers and risk managers promoting a One Health approach against public health threats.

## 1. Introduction

The increasing occurrence of zoonoses, and recently the COVID-19 crisis highlighted the importance of having close links established between surveillance programmes in humans and animals to guide operational decision-making and serve appropriate risk management. Through the collection and analysis of temporal and spatial data on health events, surveillance is a cornerstone for guiding mitigation measures and for early detection of worrying trends, hence ensuring optimal management. This last decade, international organisations have advocated for an integrated approach of surveillance, so called One Health approach, for dealing with public health threats at the nexus of the human, animal, food and environmental sectors; this especially applies to antimicrobial resistance (AMR) (1–3).

In 2015, the World Health Organization (WHO), through its Advisory Group on Integrated Surveillance of Antimicrobial Resistance (AGISAR) published a guideline with basic information required to establish an integrated surveillance of AMR, including antibiotic use in humans, food-producing animals and retail food (4). More recently in October 2022, the publication of the “One Health joint plan of action” by the Quadripartite Organizations (FAO, UNEP, WHO, and WOAH) strengthened the One Health approach with the full integration of environmental challenges, and provided a formal and legal framework to tackle complex health challenges such as AMR at the human, animal, and ecosystem interface (3).

Collaborations are the primary feature of integration. They are considered as an interprofessional process by which surveillance programmes actors address together an issue with members of the team respectfully sharing knowledge and/or resources (5). Collaboration can occur at any step of the surveillance process, from the governance to the implementation of operational surveillance activities (e.g., sample collection, data analysis) (5). However, little is known about the factors that can foster collaborations, especially between the various surveillance programmes composing a surveillance system (6–8). A recent study pointed out that the French antimicrobial resistance surveillance system was resourceful and varied yet complex and fragmented, involving 48 surveillance programmes [targeted the human (*n* = 35), animal (*n* = 12), food (*n* = 3) and/or the environment (*n* = 1) sectors] from different domains [AMR, antibiotic use (AMU) and antibiotic residue] (9). Furthermore, collaborations among several programmes were observed, including cross-sectoral collaborations [among human (hospital and community), animal, food or environment sectors] and cross-domain collaborations (AMR and AMU). This first descriptive study indicated that the French surveillance system could be appropriate to explore reasons for collaborations.

Hence, the aim of this study was to investigate factors influencing collaborations between surveillance programmes for antimicrobial resistance in France. Ultimately, this work aimed to provide a better understanding of actors’ motivations and influences driving integration between surveillance programmes, to help researchers and risk managers promoting a One Health approach.

## 2. Materials and methods

### 2.1. Study design

We carried out a qualitative study, based on semi-structured interviews with coordinators of surveillance programmes (actors in charge of the programme with a representative role) and key-informants (experts in French AMR surveillance), to investigate the factors for the set-up of collaborations between surveillance programmes within the AMR surveillance system in France. Based on the previous identification of all AMR surveillance programmes and the description of the collaborations in place by Collineau et al. (9), coordinators of all domains (AMR, AMU, antibiotic residues) and sectors [human (hospital and community), animal, food and environment] were interviewed. Coordinators were interviewed on every single collaboration in which they were involved. Coordinators, whose surveillance programme(s) was not involved in a collaboration, were also interviewed. Moreover, in order to ensure broad investigation of factors and to cross-validate opinions, key-informants were interviewed. The eligibility criteria of key-informants were based on their expertise in AMR surveillance, their awareness of collaborations in place and their implication in the structuration of the French AMR surveillance system. The key-informants were selected through snowball sampling (both programmes coordinators and selected experts provided referrals for this recruitment).

### 2.2. Data collection

The selected participants were contacted individually by email to provide information on the study (purpose, nature, background) and were informed that their opinions and speech would remain anonymous, and that any material potentially leading to individual identification would be removed. Written consent to be part of the study was obtained ahead of the interviews.

In order to maximise both the quantity and quality of data collected, an interview guide, specific for each type of participants (coordinators versus key-informants), was drafted following the framework of the Theory of Planned Behaviour (10). The guide was pre-tested through an exploratory interview with a first coordinator. Addressed topics are presented in Table 1. The questions of the interviewer changed to delve into participants’ individual responses and to adapt to the type of surveillance programme.

**Table 1:** Topics and underlying topics of discussion during the interviews.

**Topic**	**Underlying topics**
Opening questions	Description of the surveillance programme and its role in the surveillance system (for coordinator) Description of their role in the AMR surveillance system and its integration (for key-informant)
Decision to take part in a collaboration	Factors involved in the implementation of collaboration Presentation of the decision-making process Role-players involved/people influencing the decision Evolutions regarding the decision to collaborate
Perception and opinion on the collaboration	Purpose of the collaboration Opinion and view on the organisation and the management of the collaboration Impact of collaborative activities on the surveillance programme Relationships with other actors Outputs and outcomes of the collaboration Expectations regarding the collaboration
Motivation and interest behind participation in a collaboration	Factors that influenced participation Personal interests Third-party opinions or arguments that influenced the decision
Drawback and obstacle for participating	Factors that influenced refusal to collaborate Reasons for dissatisfaction Changes in viewpoint
Impact of the collaboration	Benefits for surveillance programmes involved Added value for other actors
Closing questions	General feeling on the French surveillance system of antimicrobial resistance and its *One Health-ness* Any further elements

Given the travel restrictions related to the COVID-19 pandemic, all interviews were conducted remotely, by videoconference, using Microsoft Teams^®^ software. In order to ensure the comparability of the information collected, one of the interviewer (L. R.) was systematically present at all the interviews and was assisted by one or two other interviewers (other co-authors) depending on the number of people interviewed. Interviews with key-informants were systematically individual ones, whereas the number of respondents for the interviews with coordinators varied from one to four, depending on the main coordinator’s willingness to be accompanied by other co-coordinators (from the same programme).

At the beginning of the interview, the aim and background of the study were explained, as well as the interview’s confidentiality rules, and the roles of the respondents were collected. Although the interviewers used interview guides, respondents were free to introduce any other information they felt was relevant. Interviews were recorded to facilitate the dialogue and subsequent analysis. The interviewers’ notes were shared among the co-authors after each interview. Data continued to be collected until saturation occurred (i.e., a point where collecting more data would not lead to new information related to the research questions) (11).

### 2.3. Data analysis

**Figure 1:**
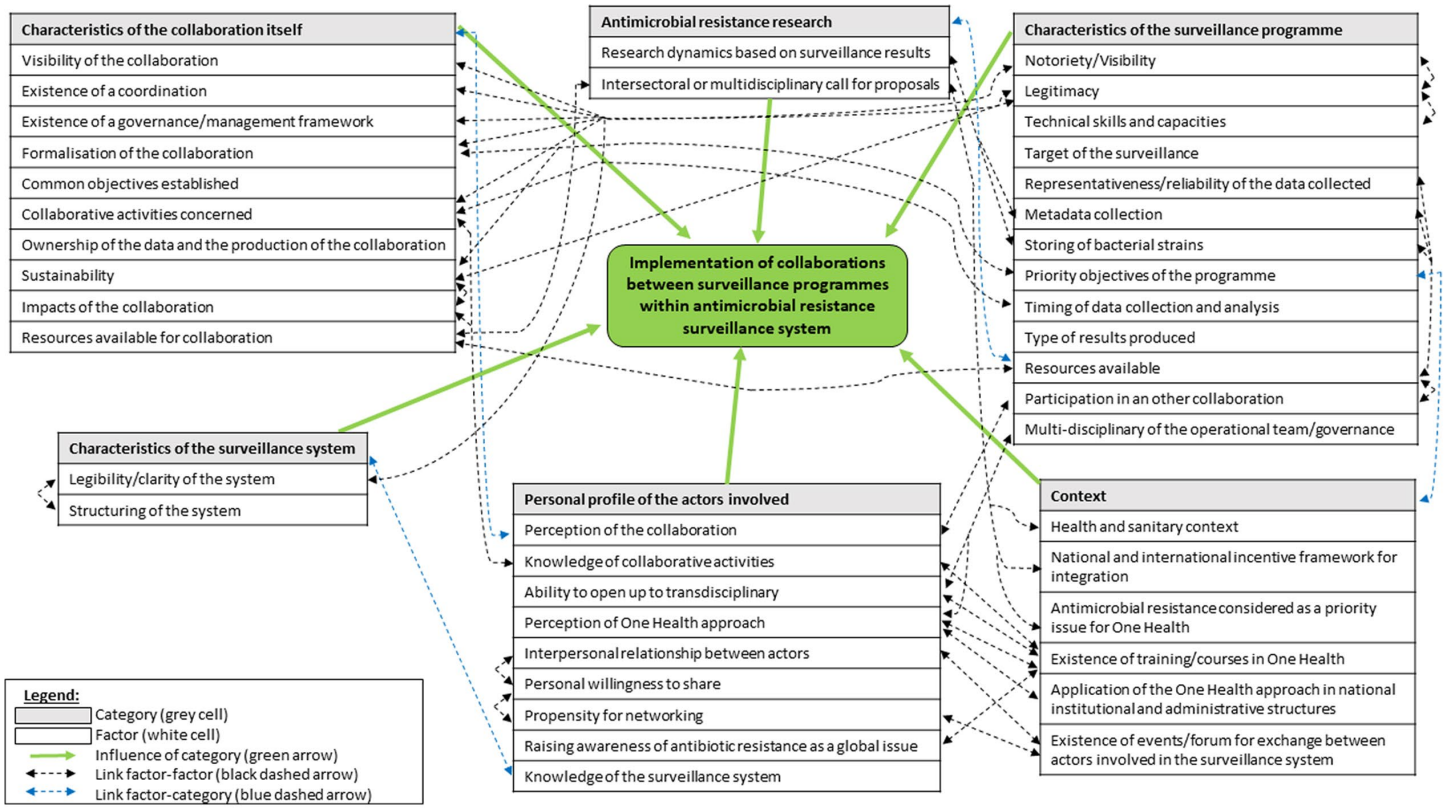
Map demonstrating the relationships between factors and categories of drivers mentioned by respondents as influencing collaborations between AMR surveillance programmes.

The interview recordings were manually transcribed and compiled with the notes. At first, data analysis involved reading through all of the transcripts to get a sense of the dataset as a whole (12). Then, the transcripts were subjected to thematic analysis, as described by Beaud and Weber (13). Specifically, thematic analysis is a method of examination of the content of discourses to identify, analyse and interpret meanings gathered in themes. The analysis was conducted inductively in a circular process and used a constant comparative method (14): repetitions of forward and backward movements from transcripts, gathering of text fragments, attribution of codes and introduction of inferences (11). Before making any inference, evidence to the contrary was sought. The data were examined in regard to the research questions, significant text fragments were identified, coded and grouped into categories, i.e., groups of content that share common feature. Similarly, categories were organised around themes. When a collaborative factor was identified to be linked with another one (i.e., mentioned together), this link was search for in other interviews. Factors were considered as mutually dependent once cross-validation was achieved (links in Figure 1). The triangulation principle (i.e., cross-checking information to validate each inference) and iteration principle (i.e., looking for repeats and synergy in transcripts) were strictly applied (15). To protect respondents’ confidentiality, all results in this paper were anonymized. Note that all verbatim quotes cited in this paper have been translated from French (Supplementary Table).

### 2.4. Ethical statement

Since this qualitative study was not a clinical trial, it did not require the formal consent and approval by the ‘Comité de protection des personnes’ in France (French ethics committee). Nevertheless, this research followed ethical rules in compliance with the Statement of Ethical Practise of the British Sociological Association (16), and was validated by the legal affairs department of the French Agency for Food, Environmental and Occupational Health and Safety (ANSES).

## 3. Results

In total, 51 semi-structured interviews were conducted for 68 participants, including 53 coordinators and 15 key-informant experts (Table 2). Some of the interviews with coordinators were multi-participants (14 interviews out of 36), whereas all interviews with key-informants were solo. Interviews were performed from March to June 2021 and lasted between 27 and 119 min (median: 57 min). Four of the participants were coordinating two surveillance programmes and, therefore, were interviewed about both programme simultaneously. In total, we collected points of views of 53 (co-)coordinators representing 40 surveillance programmes, among 48 operational surveillance programmes of AMR in France in 2021. We interviewed all coordinators, whose surveillance programme(s) was involved in a cross-sectoral or cross-domain collaboration in 2021 (Supplementary Figures 1, 2).

**Table 2:** Characteristics of the 51 interviews.

Type of actors	Number of interviews	Number of actors interviewed^*^	Length of interview in minutes: median [min; max]
Coordinator of surveillance programme	36	53	59 [27; 90]
Key-informant expert	15	15	55 [41; 119]

* From 1 to 4 coordinators interviewed per session.

Thematic analysis revealed three major themes corresponding to the two research aims (Table 3). Results are presented according to this frame. Note that there were no differences between the views of the respondents from the different sectors or of coordinators involved in collaborations or not.

**Table 3:** Overview of the research aims linked to the themes and categories that emerged during data analysis.

**Research aim**
**Theme**
**Categories**
To improve understanding of the factors that influence the implementation of collaboration between surveillance programmes	Key to setting up collaboration and actors influences	Personal profile of actors involved and importance of interpersonal relationships	A fragmented surveillance system with a lack of legibility	Characteristics of the surveillance programmes influencing collaborations	Collaborations’ characteristics influencing collaborations	Antimicrobial resistance research as a springboard for collaborations	The influence of the broader context	Impacts of collaboration	Benefits of collaboration at different levels	A necessary balance between collaboration and stand-alone existence
To explore challenges of the One Health approach for the surveillance system	Perception of the One Health approach	Plural visions of a theoretical approach that is difficult to grasp	A need for engagement of diverse stakeholders	A need for common indicators

### 3.1. Key to setting up collaboration and actors influences

Among the reasons for and limits to setting up collaborations, our study identified 42 factors grouped into six categories (Figure 1). The Figure 1 illustrates that most factors were linked together, according to the respondents.

#### 3.1.1. Personal profile of actors involved and importance of interpersonal relationships

Thematic analyses revealed that collaborations depended on the profile of actors involved, especially their willingness to share their data or expertise, to open up to transdisciplinary approaches, their perception of the One Health approach, as well as their awareness of antimicrobial resistance as a global issue. Moreover, all respondents reported that a history of good quality relationship between actors fostered the implementation of collaborations between surveillance programmes. These interpersonal relationships were formed because people had common educational backgrounds, used the same disciplinary language or worked in the same sector or on the same pathogens. This appeared to lead to a trustful relationship between them, which facilitated exchanges, mutual understanding, and thus collaboration. Therefore, collaborations were first based on people relationships and second on shared sectors or disciplines.

“*They are people we have known for years and with whom we get on well. The collaboration between us is natural*” No. 1.

*“I would say that it is more collaborations based on individuals, than collaborations let's say of organisations”* No. 44.

Respondents reported that implementation of collaboration between different surveillance sectors, or between coordinators of different disciplines was difficult because of the lack of knowledge of the people involved, who felt that they did not share the same issues.

*“The coordinators of programmes A and B are people […] who are in the field of public health epidemiology. In programme C, they are pure microbiologists. […] And so on the one hand it's the long view on a few pathogens and on the other hand a much more transversal vision. That's perhaps why it's difficult to get them to communicate!”* No. 17.

Respondents mentioned that knowledge and interpersonal relationships were mainly achieved through networking at scientific events. Thus the propensity of coordinators to network, as well as the existence of events facilitating exchanges between actors, influenced the implementation of collaborations.

*“Through this symposium, which takes place every two years, we share our work and it is an opportunity to forge links and collaborations”* No. 7.

Among the potential collaborative activities for surveillance, respondents were more frequently engaged in joint dissemination of the results, than in governance or other operational activities (e.g., design of the surveillance protocol, data collection, or data analysis). Furthermore, several respondents (about one-third of the coordinators) only envisaged joint communication of the results between surveillance programmes, due to a lack of awareness of other possible collaborative activities, which limited the scope of collaborations.

*“We don't work on the same bacteria at all. […] They work on bacteria* A *and they work on bacteria* B*, so there you go. And so we can't collaborate with them”* No. 40.

#### 3.1.2. A fragmented surveillance system with a lack of legibility

The respondents did not have a good knowledge of the antimicrobial resistance surveillance system in France, of other surveillance programmes existing in other sectors or domains, and, subsequently, of what the latter could bring to them in the framework of collaboration. According to them, this lack of knowledge was linked to the complexity of the French surveillance system, which was fragmented and lacked legibility (numerous programmes, and all the surveillance programmes were not known by stakeholders) (9). In addition, the surveillance system was perceived as being very sector-based, impacting working habits and collaborations, that were primarily set up within the same sector (e.g., human health) or within the same surveillance domain (e.g., antibiotic consumption).

*“It's really hard to get people to work together because of the number of programmes and also because of corporatism. I find that the world of human health is really many, many silos, with people who don't talk to each other, with different labels”* No. 15.

#### 3.1.3. Characteristics of the surveillance programmes influencing collaborations

Thematic analysis revealed that 13 factors related to the characteristics of the surveillance programmes influenced collaboration (Figure 1).

##### 3.1.3.1. Structural aspects

Regarding the mechanism of implementation of collaboration, we observed that they were organised according to sectors and domains (surveillance targets) of the programmes. Indeed, either the surveillance programmes were in the same sector (e.g., human health) and collaborated because their surveillance domains were different (resistance in different settings, or antibiotic use versus resistance), or the collaborating programmes focused on the same domain but in different sectors (e.g., enterobacterales in human and animal health).

Additionally, it was interesting to note that the existing collaborations supported the development of new collaborations. In particular, the participation of programmes to national or supranational networks (usually well recognised), placed them in a dynamic that made the coordinators more inclined to collaborate.

*“In the scientific committee of subsystem X, we will actually exchange in terms of methodology, or participate to studies between surveillance programmes […] We have sometimes collaborated within the subsystem itself, on particular themes that are of interest to us”* No. 34.

##### 3.1.3.2. Operational aspects

First, programmes with notoriety and legitimacy (via mandatory surveillance, national recognition, or long history of existence) were those mainly involved in collaborations. Moreover, surveillance programmes known to collect good quality data (good representativeness, large coverage), or metadata (such as geographical indication, socio-demographic or clinical information on patients), or to store bacterial strains were more engaged into collaborations. Indeed, these characteristics allowed them to compare their data more easily or relevantly between each other, or to do more in-depth analyses with other programmes with specific resources (e.g., WGS). Indeed, our study showed that for establishing joint analysis and valorization of surveillance data, it was necessary for the programmes involved to have comparable methods, compatible data collection and analysis timelines, and similar geographical coverage.

*“With programmes A and B you have an idea of the prevalence of resistance, but you don't know which strains are circulating. So necessarily there are these collaborations, there have to be shipments of strains to programme C, because it has the expertise in characterising the strains so that we can tell which clones are circulating”* No. 9.

#### 3.1.4. Collaborations’ characteristics influencing collaborations

The impact of collaboration, particularly in terms of benefits for actors or surveillance programmes, emerged as a key element in the implementation and sustainability of collaborations. While collaborations were first established based on interpersonal links or informal relationships, the coordinators were more inclined to collaborate if the collaboration was then structured and formalised (with an agreement or a charter for example). This formalism helped to reassure the actors involved. Moreover, respondents reported that the existence of coordination, of a governance or management framework for collaboration, and the formal definition of common objectives between surveillance programmes also influenced the establishment of collaborations.

*“Everyone found their interest in it. We drew up a charter, of course! We were very careful as we wanted it to be very respectful; there is a charter of commitment, and of rights and duties of each participating surveillance programme”* No. 36.

The thematic analysis also revealed that the type of collaborative activities implemented also influenced the setup of collaborations. Collaborative activities with high visibility (e.g., external joint communication) increased both visibility and legitimacy of surveillance programmes and fostered collaborations. Finally, coordinators reported that the visibility of the collaboration itself influenced the implementation of collaboration. In fact, coordinators had interest in implementing it, since its visibility contributed to both the reputation and legitimacy of the surveillance programmes involved, and it also created the desire for other programmes to collaborate.

#### 3.1.5. Antimicrobial resistance research as a springboard for collaborations

Respondents indicated that cross-sectoral or multidisciplinary calls for research projects encouraged collaboration between surveillance programmes. It was also a way of obtaining the resources necessary for initiating a collaboration. Respondents indicated research projects could thus be the first step to kick start more permanent collaborations between surveillance programmes.

*“We have already done several research projects with programme A […] We have to continue to move in that direction. It's not yet an organised routine activity, if you like. It's taking shape more around research projects, which are in essence on-offs, than by something in continuous flow. I think we need to move towards this now. This is an essential aspect of this One Health approach”* No. 2.

More broadly, research was seen as a means of enhancing the value of the collaborations and programmes involved (e.g., publications reinforced the reputation of programmes and coordinators). Research also supported collaborations as it helped to improve surveillance and thus strengthen surveillance programmes that were then better able to collaborate. Ultimately, the thematic analysis showed that the dynamics of research that built on the surveillance data produced by the surveillance programmes was a factor for collaboration.

#### 3.1.6. The influence of the broader context

According to the respondents, the broader health context (including the COVID-19 pandemic) played a role in the establishment of collaborations, influencing both the surveillance priorities and the resources allocated for these collaborations. Moreover, collaborations between surveillance programmes were strengthened when antimicrobial resistance was considered as a One Health priority by coordinators and funders. More generally, collaborations were enhanced by various elements that fostered integration, such as a national or international framework to support integrated surveillance systems, the application of the One Heath concept in institutional or administrative bodies, and the existence of specific One Health trainings in the university or academic curricula of coordinators.

*“The Covid crisis has helped quite a bit in terms of awareness of the interconnection between human and animal health and the health of ecosystems […]. The topic of antimicrobial resistance should benefit from this general "One Health" impetus, even if at first glance it may seem to suffer a little from it. Because emerging infectious diseases have come to the forefront, and antimicrobial resistance, which was considered the number one threat in the "One Health" field, has taken a back seat”* No. 30.

### 3.2. Impacts of collaboration

#### 3.2.1. Benefits of collaboration at different levels

According to the respondents, the benefits of implementing collaborations were diverse and occurred at various levels (Table 4). For example, for the surveillance programmes involved, the collaboration led to an increased efficiency in surveillance through the pooling of resources, such as the sharing of experience or expertise between coordinators. While collaborations were primarily based on quality and trusting relationships between actors, it was interesting to note that collaborations also helped to strengthen the link between them.

**Table 4:** Benefits identified from collaboration within the surveillance system and associated level.

Level	Benefit
Surveillance programme	Pooling of material, human and financial resources for surveillance activities and deliverables (reduced surveillance costs) Broadened range of surveillance activities Improved surveillance (reactivity, accuracy, etc.) Increased visibility Strengthened legitimacy Improved sustainability of surveillance programme
Surveillance system	Greater coherence Efficiency gain – optimisation of surveillance (improved and harmonized methods, timeliness, reactivity etc.)
Actors involved in surveillance	Strengthened professional network Strengthened interpersonal relationships Increased awareness regarding antimicrobial resistance Gain in skills or expertise Better understanding and knowledge of other disciplines and sectors Gain in reputation
Public interest	Expanded scientific knowledge Improved prevention and control strategies

*“That's it, ideally collaborating would be to build stronger and longer lasting connections with people from the programme A”* No. 7.

#### 3.2.2. A necessary balance between collaboration and stand-alone existence of surveillance programmes

The availability of resources was one of the major factors for the implementation of collaborations and was mentioned by almost all respondents. For a collaboration to work and be sustainable, specific resources (time and funding) had to be found or allocated from the budgets of the programmes involved. However, it emerged from the interviews that the majority of surveillance programmes were operating on a just-in-time basis with limited or even insufficient resources to maintain high quality of their own data collection or analysis. Consequently, the set-up of collaborations appeared to be of secondary importance, compared to maintaining the operations of their own surveillance programme.

*“It's very difficult to set up collaborations with the budget we are currently being allocated”* No. 2.

*“People are quite willing to collaborate in both directions, so that's really good. The big difficulty is the priorities of each programme, and therefore the time allocated to this collaboration”* No. 18.

Paradoxically, even if the pooling of resources enabled by collaboration was perceived as a potential benefit, it did not counteract the view of the respondents that collaborative activities came only after the surveillance programme’ own activities. However, an exception occurred when collaboration was included in the priority activities of the programmes (three programmes). By formalising them in this way, collaborations were more legitimate and their funding was simpler.

*“It is certain that the fact that it is in our mandate encourages us to set up [collaborations] and encourages us, probably in a subjective way, to carry out collaborations”* No. 44

For the respondents, in essence, the collaboration should bring an added value for each of the parties, comparable to a win-win approach between programmes.

*“Putting together these data, juxtaposing them, making them talk, while respecting the surveillance programmes, was an extremely strong motivation! Because for each member it was a demonstration that they existed, it valued them”* No. 36.

While collaboration was a way to gain visibility and legitimacy, it was also seen by some respondents as a threat to the visibility or sustainability of the programmes involved, if they were to give way to the collaboration itself or to one of the parties.

*“It was a bit complicated. They had constraints, which we can understand. Because of the fear that the surveillance programme A would completely absorb the programme B. There was already a need to really clarify the collaboration”* No. 44.

Finally, it was interesting to observe that some programmes, far from positioning as collaborators, saw themselves more as competitors in the search for funding or in responding to project calls (the latter remaining mostly sectoral or monodisciplinary).

*“I guess that it's not very easy to get programmes to work together, except for those that already work very well, because for personal or friendly reasons they work together. But there is quite a lot of competition eh, for surveillance programmes!”* No. 17.

### 3.3. Perception of the One Health approach

#### 3.3.1. Plural visions of a theoretical approach that is difficult to grasp

It emerged from the interviews that the One Health approach appeared difficult to grasp in a concrete way and remained a relatively abstract notion. Firstly, it appeared that the One Health approach was difficult to translate and explain. Secondly, the respondents had different visions of it, ranging from complete integration between all sectors up to minimum integration to improve human health only. As a consequence, there were differences in orientation of coordinators towards what One Health means for their surveillance programme.

*“The One Health for me is a concept, alright, that I would say a little bit of a facade. What may be behind it seems much less clear to me”* No. 17.

*“There are two ways of conceiving One Health. There are those who say: ‘One Health is human health to which other healths must contribute’, which is the very medical approach of One Health, very anthropocentric. And then there are those who say: ‘No, One Health is putting the three sectors at the same level of importance, because the poor health of one will influence the health of the other two in any direction’”* No. 12.

#### 3.3.2. Need for engagement of diverse stakeholders

According to the respondents, for the One Health concept to become a reality in surveillance of antimicrobial resistance, it should not only be implemented by all actors at different levels of organisation (ministry, administration, university, research centre, laboratories, etc.), but also supported by all actors in the system (risk managers, researchers, teachers, etc.). All the respondents testified that there is room for improvement in the application of this concept for antimicrobial resistance surveillance.

*“We do not have the impression that there’s anything there. There’s not something integrated between the animal and the human sectors. At a time when we are very One Health, I think we could do better. Already we depend on two different ministries, that does not help I think”* No. 9.

It was interesting to note that, according to the respondents, the impetus for the One Health approach in antimicrobial resistance surveillance currently comes mainly from surveillance programmes (where operational or governance teams are multidisciplinary, for example) or from the academic and scientific world (via the organisation of interdisciplinary or cross-sectoral events, which make it possible to create interpersonal relationships). They mentioned that this impetus should also come from transdisciplinary education or training of actors so that they better understand each other.

*“It's complicated to structure a One Health team locally, you have to get it accepted! In other words, they tell you that you are scattered. And just in a single team, try to integrate a sociologist, you'll see!”* No. 13.

*“That's the limit of One Health, in fact, you want to integrate everything and at the same time you're not ready to understand everything […] They [the disciplines] don't have the same language: when a sociologist talks to me I don't understand anything, and I think he's going to smoke me out”* No. 25.

Besides, the respondents highlighted the importance, in the short term, to implement a national coordination of integrated surveillance, via, for example, the creation of a cross-sectoral operational team with dedicated resources. They also emphasised the importance of transdisciplinary and cross-sectoral training and education. The latter could contribute both to acculturate the actors to this approach and to foster links between coordinators from different sectors.

*“One way of improving this is to take the problem at its roots and create a common core of training […] Eventually, if the vets, pharmacists, doctors, in short, if all these people meet in a form of common training, friendships will be created and people will follow each other, and perhaps, in addition, there is a common understanding”* No. 42.

*“Beyond surveillance, it is a more general issue, and we have asked for this several times without success, that there really is an interministerial delegate for antimicrobial resistance who has authority over the ministries to obtain results […] In terms of showing the importance of the topic it would be a positive signal”* No. 30.

Finally, several respondents (a third of all respondents) regretted that the One Health global approach was insufficiently considered as a prerequisite and was still too often taken into account only at the very end of the process.

*“In fact, during specialty training, we already try to teach them their own specialty and we consider that this One Health topic is a luxury”* No. 18.

#### 3.3.3. A need for common indicators

According to the respondents, a concrete way to implement the One Health approach would be to have a few common indicators across sectors, which all surveillance programmes could calculate, in order to make all collected data interoperable. These indicators should be simple, operational and relevant to be used widely.

*“That's typical for the prescription data, it would be good to have an indicator in common between the small animals or the big animals and the humans at the same time. Because here we don't really understand the parallel, apart from the direction of the trends, well, between the ALEA [Animal Level of Exposure to Antimicrobials] and the DDD [Defined Daily Dose]”* No. 22.

However, the respondents stressed that the implementation of such indicators could jeopardise the operations of surveillance programmes should they require a profound change in the methodology used. These changes would also have to be made in agreement with programme funders (government, agency, private sector, associations, professional organisations), who do not necessarily consider the One Heath approach. In the end, in a context where major changes would be necessary, the respondents were divided between the need for either a change of practise driven by the coordinators in a collegial manner, or by a regulatory, national or supranational demand.

## 4. Discussion

To our knowledge, this study is the first to investigate levers and impediments to collaboration between antimicrobial resistance surveillance programmes. This study identified a large number of factors (that could act as incentives or barriers), gathered into six categories (Figure 1).

According to our results, actor-related factors played a decisive role in the willingness to collaborate, which can be perceived as a change in the way they implement surveillance. The process of change itself relies on the consent and commitment of coordinators, who need to acknowledge a certain legitimacy of the collaboration to accept and implement it (17). In addition, current collaborations were based more on the network of actors and good interpersonal relationships between them, than on lead institutions or a national or supranational demand for collaboration. As collaborations were built primarily on interpersonal relationships rather than on structures, coordinators who were open to cross-sectoral approaches appeared as powerful drivers for collaborations. However, fragility resulted from this interpersonal mode of operation: collaboration could fade if coordinators who were the leaders of collaboration were to leave their function. Besides, all the contextual elements (i.e., conferences, workshops, education, training) that encouraged actors from different sectors or disciplines to better know each other, understand each other, exchange and learn from other disciplines ultimately facilitated collaborations. Therefore, in order to move towards the concrete application of a One Heath approach, all initiatives aiming at bringing coordinators together should be sustainably supported to ensure that coordinators know each other and can converse regularly. In addition, the increasing development of One Health courses related to AMR should be encouraged. The study also highlighted that coordinators lacked knowledge of the surveillance system, the existing programmes, and the role of each actor. To help in this direction, we previously published a comprehensive mapping and characterisation of all the programmes that constitute the French surveillance system (9); this was lacking so far. We are confident this work will contribute to improve the understanding of the surveillance system, hence facilitating the potential kick-off of new collaborations.

Over the last decade in France, cross-sectoral or One Heath scientific events on antimicrobial resistance that brought together programme coordinators from different sectors or disciplines have been limited. Each year during the World Antimicrobial Awareness Week, a cross-sectoral conference gathers the different ministries and public health agencies involved in surveillance, but programmes’ coordinators are no necessarily invited. In November 2021, two large French meta-networks, PROMISE and ABRomics funded through the French Priority Research Programme on antimicrobial resistance (18), were launched. They constitute an excellent opportunity to foster knowledge exchange, and improve synergies between programmes. The meta-network PROMISE aims to build a One-Health community of actors on antimicrobial resistance; it includes a data warehouse to share surveillance data between programmes, the identification of common indicators as well as pilot studies with joint data analysis. To overcome possible limitations to data sharing (e.g., data ownership regulations, internal programme rules), data can be anonymised or even aggregated for analysis at different scales. In addition, the meta-network ABRomics aims to build a One Health cross-sectoral online platform to facilitate sharing of bacterial (meta)genomics data among researchers from different sectors and disciplines, hence fostering collaboration between surveillance communities. Furthermore, at the European level, the European research agenda is moving towards a One health approach - in the steps of the One Health European Joint Programme (OHEJP, 2018–2022) – encouraging transdisciplinary research, innovation, surveillance, both at national and European levels (19). While the context is increasingly favourable to holistic approaches, Benedetti et al. (20) stressed the importance of largely promoting and communicating joint actions and transdisciplinary activities, to ensure that they are known and useful to both the scientific community and policymakers.

In terms of barriers to collaboration between programmes, the lack of human, financial and/or technical resources dedicated to collaboration, the siloed surveillance system, and the sectoral priority of programmes, appeared as challenges difficult to overcome. Moreover, the poor legibility of the surveillance system led to a lack of knowledge of existing programmes by the coordinators. In this context, the work of characterising and mapping surveillance systems appears particularly useful to support synergies between programmes (9). Although calls for proposals are a way for programmes to join forces to obtain resources, they can also encourage competition between them, and subsequently deteriorate the relationships among coordinators. This highlighted the need for intersectoral calls for proposals, with the selection of projects based on collaboration between research teams from different domains and/or disciplines to ensure a comprehensive approach of a scientific issue, and thus increase collaboration between surveillance programmes. Even if collaborations could be seen as a pooling of resources, they were still largely considered as a costly additional activity, since collaborative activities were not necessarily in line with the sectoral objectives of the programmes. Thus, the inclusion of intersectoral collaborative activities within the objectives of the surveillance programmes could improve alignment between resources and objectives, and support the development and sustainability of collaboration. All these elements underlined the need for an impetus for One Health to be given at different levels to become a reality, and not just by coordinators, but more broadly by programme funders and risk managers. According to our results, a context favourable to intersectoral/interdomain collaboration is crucial to encourage One Health collaborations. It implies that collaboration should be an intrinsic objective of surveillance, to overcome structural and operational barriers that cause difficulties to collaborate. The activities of surveillance programmes (and therefore collaborations) mainly depend on the orientations given by their funding bodies. To support the development and sustainability of collaboration, it is therefore essential that the collaborative activities are in line with the programmes’ own objectives.

In addition, administrative structural barriers between ministries of Health, Agriculture and the Environment are obstacles to progress to One Health (6, 19). More exchanges and coordination between ministries (through meetings, joint working programmes, or even the setup of an interministerial coordination body) could foster integration between siloed directorates of ministries. At the European level, a recent study highlighted that the research needs should be defined from a One health perspective, requiring the involvement of European Union agencies and including both policy cooperation and transdisciplinary coordination, similarly to the OHEJP approach (19). The authors also underpinned that fragmented research activities and in-silo regulations limit transdisciplinary and interagency cooperation, requiring a more horizontal approach to regulatory frameworks to fully integrate the One Health principle.

This study also enabled us to identify numerous benefits to the setup of collaborations (Table 4). Several of them were consistent with previous results dealing with the impact of integrated surveillance (6–8) and of One Health networks (21). These authors also identified the improvement of scientific knowledge, in particular a better understanding of transmission routes across sectors, the identification of the relative importance of the different reservoirs in the emergence and maintenance of resistance in humans, the identification of correlations between antibiotic use and resistance within and between sectors, and the assessment of intervention impacts within and between sectors. We believe that the use of qualitative approach applied to a dense system was particularly relevant to progress in the identification of benefits resulting from collaborative surveillance. Since impacts are seen as drivers for implementing collaborations, it would be interesting to further investigate and characterise the impacts and benefits of collaborations within the surveillance system, not only for programme coordinators, but also for all stakeholders involved.

Our study pursued a qualitative sociological approach, which is a valuable way of understanding the diversity and extent of opinions (22, 23). Although this approach does not lend itself to the quantification of each opinion in the broad population, nor to statistical inference, it helped to answer and understand why collaborations occur. The qualitative approach was therefore well suited for gaining insight into coordinators’ decisions making. Although ideally interviews should have been conducted individually without witnesses to facilitate expression of personal opinions, several interviews with coordinators were not individual, upon the coordinators request to supplement their responses with those from co-coordinators. While this allowed us to collect and reinforce diverse views on the factors of collaborations, it could hinder the spontaneity and exhaustivity of information provided by the respondents due to hierarchical link between them. Besides, all interviews were conducted remotely due to the travel restrictions, making it more difficult to analyse the gestures and reactions of respondents. Despite those limits, we believe this qualitative approach was a valuable way to capture novel information regarding reasons for collaboration that cannot be obtained using a quantitative questionnaire methodology. It was a necessary first step before possibly considering further quantitative research to weigh each factor.

The French antimicrobial resistance surveillance system appeared to be a particularly relevant case study to explore the reasons for collaborations. It was varied and fragmented, with numerous surveillance programmes involved or not in collaborations (9). Moreover, since AMR is a major public health concern involving four sectors (human, animal, food, and environment), three domains (antibiotic resistance, antibiotic use or consumption, and residue of antibiotic), several disciplines (among others epidemiology, microbiology, infectiology, hygiene, pharmacology, ecology, sociology) this system enabled us to investigate collaborations from various sights (collaborations involving two to 14 surveillance programmes). Finally, since this system was dense and complex, with heterogeneous programmes, we believed it was a better case study to identify multiple factors for collaboration than a surveillance system focusing on one particular disease.

By following analysis and sampling rules (triangulation, iteration and saturation) and thank to the confidentiality of interviews ensuring the trustworthiness of respondents answers (11, 23), we were able to identify relevant factors for collaboration between AMR surveillance programmes at the French level. Since most factors were neither specific to the French context nor the antimicrobial resistance threat, we believe these results could be useful to other collaborative surveillance systems dealing with other diseases, in other countries. However, since we were only able to identify factors of collaboration within the French AMR surveillance system, it would be interesting to perform similar studies for other surveillance systems and in different contexts (for example in a non-European country) to identify other relevant factors impacting the implementation of collaboration between programmes. Considering that we focused on a particularly complex surveillance system, it would be relevant to explore and compare which collaboration factors occur in a less fragmented surveillance system. Finally, by providing incentives to foster integration and clues to understand coordinators’ positions, our findings can be of interest to any surveillance system in other countries, for researchers, programmes’ coordinators, and risk managers to move globally towards a One Health approach of surveillance.

## Data availability statement

The datasets presented in this article are not readily available because the data used for this study were obtained through semi-structured interviews of programmes’ coordinators and key informant experts. Conditions of approval (respecting the anonymity of respondents) do not allow us to distribute or make available data directly to other parties. Data analysed are included in this published article and its supplementary information files. Requests to access the datasets should be directed to lucie.collineau@anses.fr.

## Ethics statement

Ethical review and approval was not required for the study on human participants in accordance with the local legislation and institutional requirements. Written informed consent to be part of the study was obtained from the participants before interviews.

## Author contributions

CB and LR analysed the data. CB wrote the original draft. CB, LR, MC-C, and LC conceptualised, designed, performed the study, interpreted the data, read, and approved the final manuscript. All authors contributed to the article and approved the submitted version.

## Funding

This work was supported by the French Ministry of Agriculture (http://agriculture.gouv.fr) as part of the Surv1Health Project (Plan EcoAntibio2: research grant 2019-124). No additional external funding was received for this study. The funders had no role in study design, data collection, analysis, decision to publish, or preparation of the manuscript.

## Conflict of interest

The authors declare that the research was conducted in the absence of any commercial or financial relationships that could be construed as a potential conflict of interest.

## Publisher’s note

All claims expressed in this article are solely those of the authors and do not necessarily represent those of their affiliated organizations, or those of the publisher, the editors and the reviewers. Any product that may be evaluated in this article, or claim that may be made by its manufacturer, is not guaranteed or endorsed by the publisher.

## References

[1] World Health Organization, Food and Agriculture Organization of the United Nations & World Organisation for Animal Health. Antimicrobial resistance: a manual for developing national action plans, version 1. World Health Organization. (2016). Available at: https://apps.who.int/iris/handle/10665/204470

[2] FAO, WHO, CODEX. FAO/WHO/CODEX webinar on FAO and WHO activities to support monitoring and surveillance of antimicrobials resistance in the food and agriculture sectors. (2021). Available at: https://www.grease-network.org/news/fao-who-codex-webinar-for-amr-surveillance

[3] FAO, UNEP, WHO, WOAH. One health joint action plan 2022–2026 - working together for the health of humans, animals, plants and the environment. Rome: FAO (2022).

[4] Management of Emerging Risks in Southeast Asia. FAO/WHO/CODEX webinar for AMR surveillance / News - GREASE - Management of emerging risks in Southeast Asia. (2021). Available at: https://www.grease-network.org/news/fao-who-codex-webinar-for-amr-surveillance (Accessed September 30, 2022).

[5] Bordier M Delavenne C Nguyen DTT Goutard FL Hendrikx P. One health surveillance: a matrix to evaluate multisectoral collaboration. Front Vet Sci. (2019) 6:109. doi: 10.3389/fvets.2019.00109, PMID: 31106210 PMC6492491

[6] Stärk KDC Arroyo Kuribreña M Dauphin G Vokaty S Ward MP Wieland B. One health surveillance – more than a buzz word? Prev Vet Med. (2015) 120:124–30. doi: 10.1016/j.prevetmed.2015.01.019, PMID: 25722055

[7] Bennani H Cornelsen L Stärk KDC Häsler B. Evaluating integrated surveillance for antimicrobial use and resistance in England: a qualitative study. Front Vet Sci. (2021) 8:743857. doi: 10.3389/fvets.2021.743857, PMID: 34805336 PMC8596565

[8] Aenishaenslin C Häsler B Ravel A Parmley EJ Mediouni S Bennani H. Evaluating the integration of one health in surveillance systems for antimicrobial use and resistance: a conceptual framework. Front Vet Sci. (2021) 8:611931. doi: 10.3389/fvets.2021.611931, PMID: 33842569 PMC8024545

[9] Collineau L Bourely C Rousset L Berger-Carbonne A Ploy MC Pulcini C. Towards One Health surveillance of antibiotic resistance: characterisation and mapping of existing programmes in humans, animals, food and the environment in France, 2021. Eurosurveillance. (2023) 28: 2200804. 37261729 10.2807/1560-7917.ES.2023.28.22.2200804 PMC10236929

[10] Ajzen I. The theory of planned behavior. Organ Behav Hum Decis Process. (1991) 50:179–211. doi: 10.1016/0749-5978(91)90020-T

[11] Mukamurera J Lacourse F Couturier Y. Des avancées en analyse qualitative: pour une transparence et une systématisation des pratiques. Recherches Qualitatives. (2006) 26:110–38. doi: 10.7202/1085400ar

[12] Graneheim UH Lundman B. Qualitative content analysis in nursing research: concepts, procedures and measures to achieve trustworthiness. Nurse Educ Today. (2004) 24:105–12. doi: 10.1016/j.nedt.2003.10.001, PMID: 14769454

[13] Beaud S Weber F. Guide de l’enquête de terrain: produire et analyser des données ethnographiques. Paris: Éditions la Découverte (2003). 356 p.

[14] Braun V Clarke V. What can “thematic analysis” offer health and wellbeing researchers? Int J Qual Stud Health Wellbeing. (2014) 9:26152. doi: 10.3402/qhw.v9.26152, PMID: 25326092 PMC4201665

[15] Olivier De Sardan JP. La rigueur du qualitatif: Les contraintes empiriques de l’interprétation socio-anthropologique. Louvain-La-Neuve: Academia-Bruylant. (2008). p. 46–121.

[16] British Sociological Association. Statement of ethical practice for the British Sociological Association (March 2002). (2002). Available at: https://www.britsoc.co.uk/equality-diversity/statement-of-ethical-practice/#_cov

[17] Paillé P. Changement organisationnel et mobilisation des ressources humaines. Paris: L’Harmattan (2003). 258 p.

[18] INSERM. Interface nationale ANTIBIORÉSISTANCE - Projets et actions soutenu: Action 2 – Résultats des AAP structurants du PPR Antibiorésistance, 3 projets retenus 2021. (2021). Available at: https://ppr-antibioresistance.inserm.fr/fr/projets-actions-soutenus/action-2-resultats-des-aap-structurants-du-ppr-antibioresistance-3-projets-retenus/#promise

[19] Bronzwaer S Catchpole M de Coen W Dingwall Z Fabbri K Foltz C. One health collaboration with and among EU agencies - bridging research and policy. One Health. (2022) 15:100464. doi: 10.1016/j.onehlt.2022.100464, PMID: 36561708 PMC9767809

[20] Benedetti G Jokelainen P Ethelberg S. Search term “one health” remains of limited use to identify relevant scientific publications: Denmark as a case study. Front Public Health. (2022) 10:938460. doi: 10.3389/fpubh.2022.938460 35968488 PMC9368311

[21] Streichert LC Sepe LP Jokelainen P Stroud CM Berezowski J Del Rio Vilas VJ. Participation in one health networks and involvement in the COVID-19 pandemic response: a global study. Front Public Health. (2022) 10:830893. doi: 10.3389/fpubh.2022.830893, PMID: 35284359 PMC8907588

[22] Guest G Bunce A Johnson L. How many interviews are enough? An experiment with data saturation and variability. Field Methods. (2006) 18:59–82. doi: 10.1177/1525822X05279903

[23] Tracy SJ. Qualitative quality: eight a "big-tent" criteria for excellent qualitative research. Qual Inq. (2010) 16:837–51. doi: 10.1177/1077800410383121

