# Supplementary material for: How to move towards One Health surveillance? A qualitative study exploring the factors influencing collaborations between antimicrobial resistance surveillance programmes in France
Source: Front Public Health. 2023 Jul 11;11:1123189. doi: 10.3389/fpubh.2023.1123189 (PMC10367569; doi:10.3389/fpubh.2023.1123189)
Supplement: Supplementary file 1 [file Table_1.docx]

**Supplementary Table. Indicative table for translations presented in the paper: French transcribed quotes with their English translation** **(with anonymity number of respondents interviewed)**

| **French quotes** | **English quotes** |
| --- | --- |
| *«* *C'est des gens qu'on côtoie depuis des années et avec qui on s'entend bien. Les collaborations entre nous sont naturelles* *»* n° 1 | *“They are people we have known for years and with whom we get on well. The collaboration between us is natural”* No. 1. |
| *« Je dirais que c'est plus des collaborations d’individus, que des collaborations disons d'organismes »* n° 44 | *“I would say that it is more collaborations based on individuals, than collaborations let's say of organisations”* No. 44. |
| *« On prend les animateurs des programmes A et B, sont des gens [...] qui sont dans le domaine on va dire épidémiologie santé publique. Dans le programme C c'est des microbiologistes purs. [...] Et donc c'est d'un côté la longue vue sur quelques pathogènes et de l'autre côté une vision beaucoup plus transversale. C'est peut-être pour ça d'ailleurs qu'il est difficile de les faire communiquer hein ! »* n° 17 | *“The coordinators of programmes A and B are people [...] who are in the field of public health epidemiology. In programme C, they are pure microbiologists. [...] And so on the one hand it's the long view on a few pathogens and on the other hand a much more transversal vision. That's perhaps why it's difficult to get them to communicate!”* No. 17. |
| *«* *Au travers de ce symposium qui a lieu tous les deux ans on partage nos travaux et c'est l'occasion de nouer des rapprochements et des collaborations* *»* n° 7 | *“Through this symposium, which takes place every two years, we share our work and it is an opportunity to forge links and collaborations”* No. 7. |
| *« On ne travaille pas du tout sur la même bactérie. […] Ils travaillent sur la bactérie a et ils travaillent sur les bactéries b donc voilà. Et donc on ne peut pas collaborer avec eux »* n° 40 | *“We don't work on the same bacteria at all. [...] They work on bacteria* A *and they work on bacteria* B*, so there you go. And so we can't collaborate with them”* No. 40. |
| *« C'est vachement dur de faire bosser les gens ensemble pour à la fois des questions de nombre d’organisations, et des questions aussi de corporatisme. Je trouve que le monde de la santé humaine c'est vraiment beaucoup, beaucoup de silos, avec des gens qui ne se parlent pas, des étiquettes différentes »* n° 15 | *“It's really hard to get people to work together because of the number of programmes and also because of corporatism. I find that the world of human health is really many, many silos, with people who don't talk to each other, with different labels”* No. 15. |
| *« Au conseil scientifique du sous-système X on va échanger effectivement en termes méthodologiques, ou alors participer à des études entre dispositifs [...] On a eu parfois des collaborations à l'intérieur même du sous-système, sur des thématiques particulières qui nous intéressaient »* n° 34 | *“In the scientific committee of subsystem X, we will actually exchange in terms of methodology, or participate in studies between surveillance programmes [...] We have sometimes collaborated within the subsystem itself, on particular themes that are of interest to us”* No. 34. |
| *« Avec les dispositifs A et B vous avez une idée de la prévalence de la résistance, mais vous ne savez pas qu'elles souches circulent. Donc forcément il y a ces collaborations, il faut qu'il y ait des envois de souches au dispositif C, parce qu’il a l'expertise de la caractérisation des souches pour qu'on puisse dire quels clones circulent »* n° 9 | *“With programmes A and B you have an idea of the prevalence of resistance, but you don't know which strains are circulating. So necessarily there are these collaborations, there have to be shipments of strains to programme C, because it has the expertise in characterising the strains so that we can tell which clones are circulating”* No. 9. |
| *« Chacun y trouvait son bénef. On a fait une charte bien entendu ! On était très prudent comme on voulait que ce soit très respectueux, il y a une charte de l'engagement, et de droits et devoirs de chacun des réseaux »* n° 36 | *“Everyone found their interest in it. We drew up a charter, of course! We were very careful as we wanted it to be very respectful; there is a charter of commitment, and of rights and duties of each participating surveillance programmes”* No. 36. |
| *« Nous avons déjà fait plusieurs projets de recherche avec le dispositif X […] Il faut continuer à aller dans ce sens-là. C'est pas encore si vous voulez une activité de routine organisée. Ça prend plus corps autour de projets de recherche qui sont par essence ponctuels, que par quelque chose en flux continue. C'est ça vers quoi il faut aller je pense actuellement. C’est un aspect essentiel de cette approche One Health »* n° 2 | *“We have already done several research projects with programme A […] We have to continue to move in that direction. It's not yet an organised routine activity, if you like. It's taking shape more around research projects, which are in essence on-offs, than by something in continuous flow. I think we need to move towards this now. This is an essential aspect of this One Health approach”* No. 2. |
| *« La crise Covid a quand même pas mal aidé sur la prise de conscience de l'imbrication, des liens entre la santé humaine, animale, la santé des écosystèmes […] Le sujet de l'antibiorésistance devrait bénéficier de cet élan "une seule santé" un peu général, même si en première approche on peut avoir l'impression qu'il en souffre un peu. Parce que les maladies infectieuses émergentes sont venues sur le devant de la scène et l’antibiorésistance, qui était considérée comme la menace numéro un dans le domaine une seule santé, est passée un peu en retrait »* n° 30 | *“The Covid crisis has helped quite a bit in terms of awareness of the interconnection between human and animal health and the health of ecosystems [...]. The topic of antimicrobial resistance should benefit from this general "One Health" impetus, even if at first glance it may seem to suffer a little from it. Because emerging infectious diseases have come to the forefront, and antimicrobial resistance, which was considered the number one threat in the "One Health" field, has taken a back seat”* No. 30. |
| *« Voilà, idéalement collaborer ce serait pour nouer des liens plus forts et plus longs dans le temps avec des personnes du dispositif A »* n° 7 | *“That's it, ideally collaborating would be to build stronger and longer lasting connections with people from the programme A”* No. 7. |
| *« C'est très difficile de mettre en place des collaborations avec les enveloppes qui nous sont allouées actuellement »* n° 2 | *“It's very difficult to set up collaborations with the budget we are currently being allocated”* No. 2. |
| *« Les personnes sont tout à fait prêtes dans les deux sens à collaborer, donc ça c'est vachement bien. La grosse difficulté c'est les priorités de chacun, et donc le temps alloué à cette collaboration »* n° 18 | *“People are quite willing to collaborate in both directions, so that's really good. The big difficulty is the priorities of each programme, and therefore the time allocated to this collaboration”* No. 18. |
| *« C'est sûr que le fait que ça soit dans notre cahier des charges incite à instituer [des collaborations], pour nous incite, probablement de manière subjective, à réaliser des collaborations »* n° 44 | *“It is certain that the fact that it is in our mandate encourages us to set up [collaborations] and encourages us, probably in a subjective way, to carry out collaborations”* No. 44. |
| *« Le fait de mettre ensemble ces données, de les juxtaposer, de les faire parler, en respectant les programmes de surveillance, c'était une motivation extrêmement forte ! Parce que pour chaque membre ça a été la démonstration qu'ils existaient, ça les valorise »* n° 36 | *“Putting together these data, juxtaposing them, making them talk, while respecting the surveillance programmes, was an extremely strong motivation! Because for each member it was a demonstration that they existed, it valued them”* No. 36. |
| *« Ça a été un peu compliqué. Ils avaient des contraintes, voilà qu'on peut comprendre. Parce que par peur que le dispositif A en fait phagocyte complètement le dispositif B. Il y avait déjà la nécessité de vraiment d'éclaircir la collaboration »* n° 44 | *“It was a bit complicated. They had constraints, which we can understand. Because of the fear that the surveillance programme A would completely absorb the programme B. There was already a need to really clarify the collaboration”* No. 44. |
| *« J'imagine que ce n'est pas très facile de faire travailler ensemble des dispositifs de surveillance, sauf ceux qui fonctionnent déjà très bien, parce que pour des raisons personnelles ou amicales ils fonctionnent ensemble. Mais il y a une concurrence assez forte hein, pour les dispositifs de surveillance »* n° 17 | *“I guess that it's not very easy to get programmes to work together, except for those that already work very well, because for personal or friendly reasons they work together. But there is quite a lot of competition eh, for surveillance programmes!”* No. 17. |
| *« Le One Health pour moi c’est un concept, très bien, que je dirais un peu d'affichage. Ce qu'il peut y avoir derrière me parait beaucoup moins clair »* n° 17 | *“The One Health for me is a concept, alright, that I would say a little bit of a facade. What may be behind it seems much less clear to me”* No. 17. |
| *« Il y a deux façons de concevoir One Health. Il y a ceux qui disent : "One Health c'est la santé humaine à laquelle les autres santés doivent contribuer" qui est l'approche très médicale du One Health on va dire, très anthropocentrée. Et puis il y a ceux qui disent, "non, le One Health c'est mettre au même niveau d'importance les trois secteurs, car la mauvaise santé de l'un va influer sur la santé des deux autres dans tous les sens" »* n° 12 | *“There are two ways of conceiving One Health. There are those who say: "One Health is human health to which other healths must contribute", which is the very medical approach of One Health, very anthropocentric. And then there are those who say: "No, One Health is putting the three sectors at the same level of importance, because the poor health of one will influence the health of the other two in any direction"”* No. 12. |
| *« On a pas l'impression que là encore il y ait quelque chose, il n'y a pas un truc intégré entre l'animal et l'Homme. A une époque où on est très One Health je pense qu'on pourrait faire mieux. Déjà on dépend de deux ministères différents, ça aide pas je pense »* n° 9 | *“We don't have the impression that there's anything there. There's not something integrated between the animal and the human sectors. At a time when we are very One Health, I think we could do better. Already we depend on two different ministries, that doesn't help I think”* No. 9. |
| *« En local c'est compliqué de structurer une équipe One health, faut le faire passer quoi ! C'est à dire qu'on vous dit que vous êtes dispersés. Et juste dans une équipe, essayer d'intégrer un sociologue, vous allez voir ! »* n° 13 | *“It's complicated to structure a One Health team locally, you have to get it accepted! In other words, they tell you that you are scattered. And just in a single team, try to integrate a sociologist, you'll see!”* No. 13. |
| *« C'est la limite du One Health en fait, c'est que on veut tout intégrer et en même temps on n'est pas prêt à tout comprendre […] Elles [les disciplines] n’ont pas le même langage : quand un sociologue me parle je comprends rien, et je pense qu'il va m'enfumer »* n° 25 | *“That's the limit of One Health, in fact, is that you want to integrate everything and at the same time you're not ready to understand everything [...] They [the disciplines] don't have the same language: when a sociologist talks to me I don't understand anything, and I think he's going to smoke me out”* No. 25. |
| *« Une façon d'améliorer ça c'est de prendre le mal à la base et de créer un tronc commun de formation […] Fatalement si les vétos , les pharmaciens, les médecins, enfin si tout ce petit monde se retrouve dans une forme de formation commune, déjà il y a forcément des amitiés qui se créent et des gens qui se suivent, et peut-être en plus une culture commune »* n° 42 | *“One way of improving this is to take the problem at its roots and create a common core of training [...] Eventually, if the vets, pharmacists, doctors, in short, if all these people meet in a form of common training, friendships will be created and people will follow each other, and perhaps, in addition, there is a common understanding”* No. 42. |
| *« Au-delà de la surveillance, c'est plus général, on l'avait demandé plusieurs fois sans succès, c'est qu'il y ait vraiment, véritablement un délégué interministériel à l'antibiorésistance qui a autorité sur les ministères pour obtenir des résultats […] En termes d'affichage, en tout cas de l'importance du sujet, ça serait un signal positif »* n° 30 | *“Beyond surveillance, it is a more general issue, and we have asked for this several times without success, that there really is an interministerial delegate for antimicrobial resistance who has authority over the ministries to obtain results [...] In terms of showing the importance of the topic it would be a positive signal”* No. 30. |
| *« En fait pendant la formation de spécialités, on essaye déjà de leur apprendre leur spécialité et on considère que ce sujet One Health est un luxe »* n° 18 | *“In fact, during specialty training, we already try to teach them their own specialty and we consider that this One Health topic is a luxury”* No. 18. |
| *«* *Voilà typiquement pour la prescription ce serait bien un indicateur qu'on pourrait avoir en commun avec les petites bêtes ou les grosses bêtes et les humains à la fois. Parce que là on ne comprend pas bien le parallèle, à part le sens des courbes, enfin bon, entre l'ALEA et la DDJ »* n° 22 | *“That's typical for the prescription data, it would be good to have an indicator in common between the small animals or the big animals and the humans at the same time. Because here we don't really understand the parallel, apart from the direction of the trends, well, between the ALEA [Animal Level of Exposure to Antimicrobials] and the DDD [Defined Daily Dose]”* No. 22. |
