# Supplementary figures and images for: How to move towards One Health surveillance? A qualitative study exploring the factors influencing collaborations between antimicrobial resistance surveillance programmes in France
Source: Front Public Health. 2023 Jul 11;11:1123189. doi: 10.3389/fpubh.2023.1123189 (PMC10367569; doi:10.3389/fpubh.2023.1123189)

**Supplementary Figure 1. Profile of the key-informant experts interviewed**

**
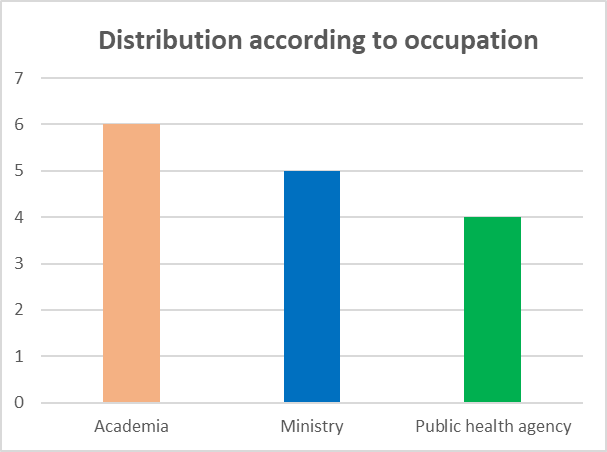

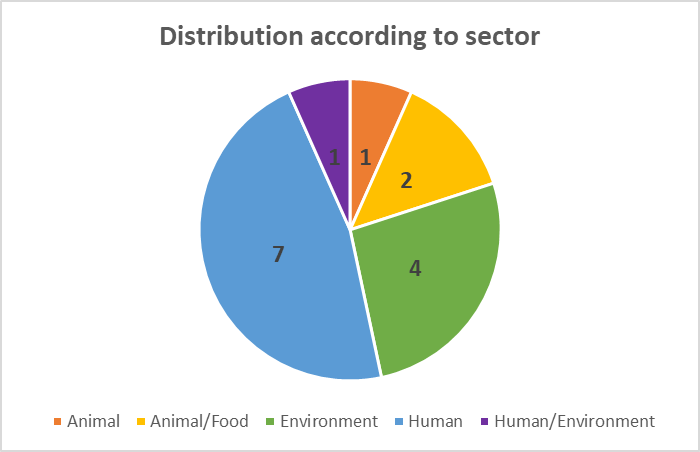
**


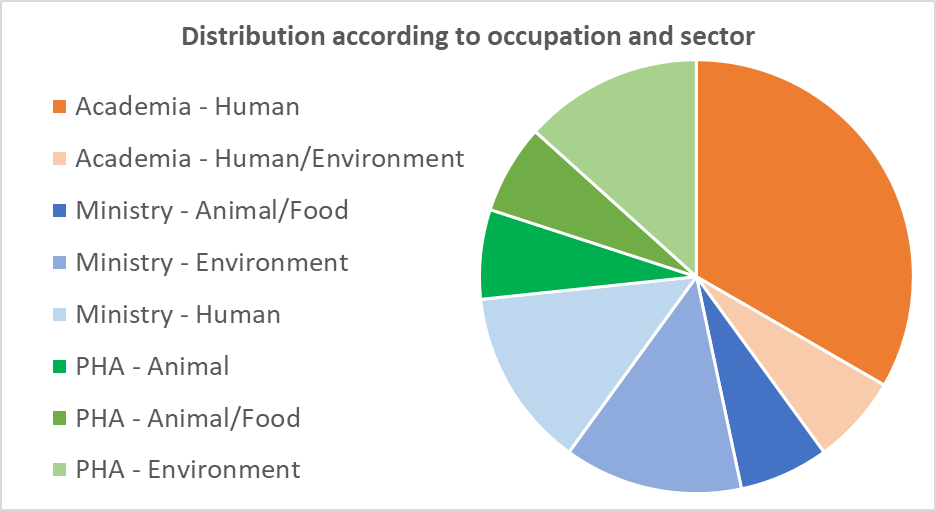

Supplement: Supplementary file 2 [file Data_Sheet_1.docx]
