# Supplementary material for: How to move towards One Health surveillance? A qualitative study exploring the factors influencing collaborations between antimicrobial resistance surveillance programmes in France
Source: Front Public Health. 2023 Jul 11;11:1123189. doi: 10.3389/fpubh.2023.1123189 (PMC10367569; doi:10.3389/fpubh.2023.1123189)
Supplement: Supplementary file 3 [file Data_Sheet_2.docx]

**Supplementary Figure 2. Characteristics of the 40 surveillance programmes coordinated by respondents**


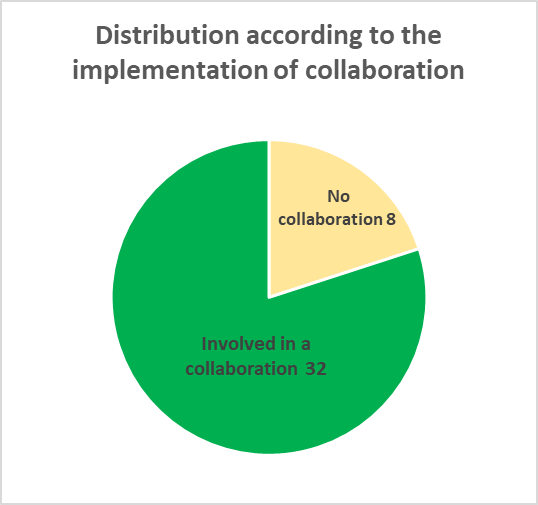
**
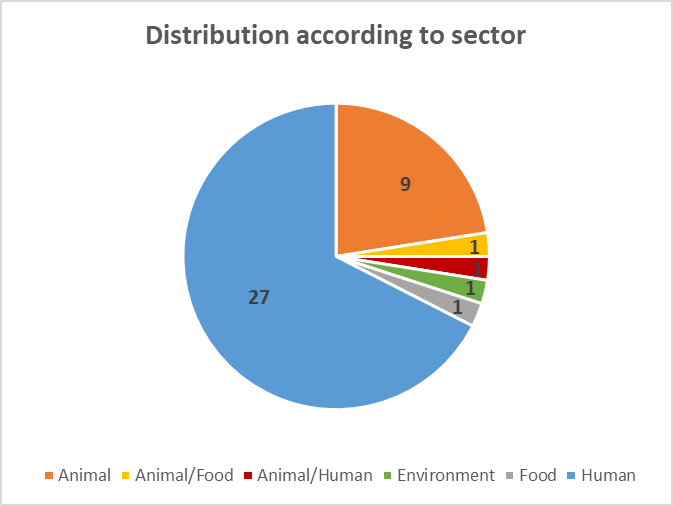
**

**
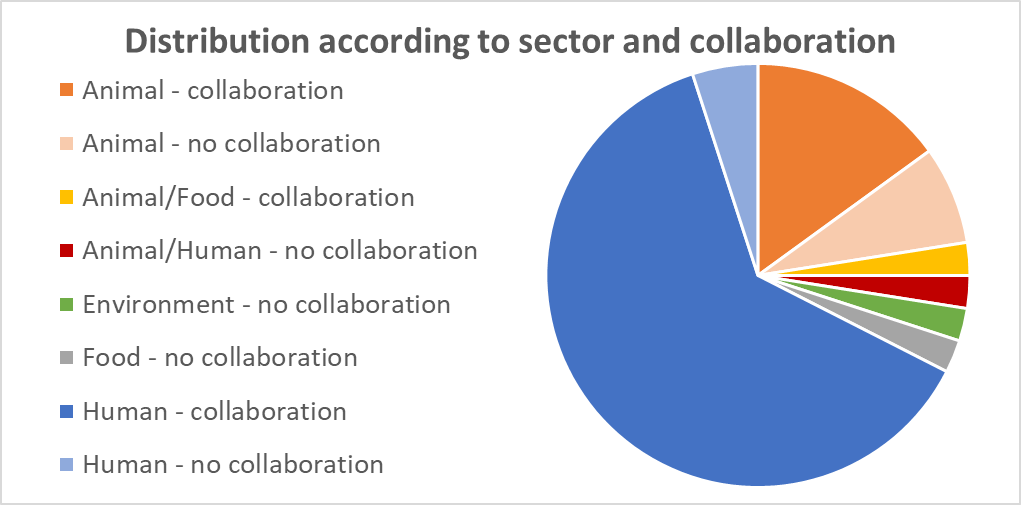
**
